# The validation of the standard Chinese version of the European Organization for Research and Treatment of Cancer Quality of Life Core Questionnaire 30 (EORTC QLQ-C30) in pre-operative patients with brain tumor in China

**DOI:** 10.1186/1471-2288-11-56

**Published:** 2011-04-22

**Authors:** Jin-xiang Cheng, Bo-lin Liu, Xiang Zhang, Yong-qiang Zhang, Wei Lin, Rui Wang, Yong-qin Zhang, Hong-ying Zhang, Li Xie, Jun-li Huo

**Affiliations:** 1 Department of Neurosurgery, Xijing Institute of Clinical Neuroscience, Xijing Hospital, People's Republic of China; 2 Department of Health Statistics, Fourth Military Medical University, Xi'an, Shaanxi Province, People's Republic of China

## Abstract

**Background:**

Health related quality of life (HRQOL) has increasingly emphasized on cancer patients. The psychometric properties of the standard Chinese version of the European Organization for Research and Treatment of Cancer Quality of Life Core Questionnaire 30 (EORTC QLQ-C30, version 3.0) in brain tumor patients wasn't proven, and there was no baseline HRQOL in brain tumor patients prior to surgery.

**Methods:**

The questionnaire EORTC QLQ-C30 (version 3.0) was administered at three time points: T1, the first or the second day that patients were hospitalized after the brain tumor suspected or diagnosed by MRI or CT; T2, 1 to 2 days after T1, (T1 and T2 were both before surgery); T3, the day before discharge. Clinical variables included disease histologic types, cognitive function, and Karnofsky Performance Status.

**Results:**

Cronbach's alpha coefficients for multi-item scales were greater than .70 and multitrait scaling analysis showed that most of the item-scale correlation coefficients met the standards of convergent and discriminant validity, except for the cognitive functioning scale. All scales and items exhibited construct validity. Score changes over peri-operation were observed in physical and role functioning scales. Compared with mixed cancer patients assessed after surgery but before adjuvant treatment, brain tumor patients assessed pre-surgery presented better function and fewer symptoms.

**Conclusions:**

The standard Chinese version of the EORTC QLQ-C30 was overall a valid instrument to assess HRQOL in brain tumor patients in China. The baseline HRQOL in brain tumor patients pre-surgery was better than that in mixed cancer patients post-surgery. Future study should modify cognitive functioning scale and examine test-retest reliability and response validity.

## Background

Cancers of the brain and nervous system account for 189,000 new cases and 142,000 deaths annually (1.7% of new cancers and 2.1% of cancer deaths) [1]. Hence, quality of life (QoL) issues are of special importance for brain tumor patients. The European Organization for Research and Treatment of Cancer (EORTC) Core Quality of Life Questionnaire (QLQ-C30) [2] and the Brain Cancer Module (BCM) [3], as well as the Functional Assessment of Cancer Therapy-Brain (FACT-Br) [4] were developed and extensively used in clinical trials. The majority of QoL instruments were developed in English and used predominantly in English-speaking populations. However in China, the study of health-related quality of life (HRQOL) is still in the developing stage. Most of QoL instruments used in China are translated from those used in developed English-speaking and western European countries. The standard Chinese version of EORTC QLQ-C30 (version 3.0) is, overall, a valid instrument to assess HRQOL in Chinese breast, gynecological, and lung cancer patients [5]; nevertheless, its reliability and validity have not yet been evaluated in brain tumor patients. To date, there is no specific brain tumor questionnaire available to assess HRQOL in China. Although HRQOL was extensively used as secondary endpoint to assess the efficacy of new treatments after surgery [6-10], the baseline HRQOL in brain tumor patients before surgery has never been investigated, especially in China.

The objects of the study were: (1) to assess the reliability and validity of the standard Chinese version of QLQ-C30 (version 3.0) in brain tumor patients in China; (2) to investigate the preoperative baseline HRQOL in brain tumor patients; (3) to compare the HRQOL discrepancies between patients before surgery and those after surgery.

## Methods

### Participants

A consecutive series of patients with either suspected brain tumor or diagnosed by MR or CT were recruited from July 2008 to December 2008 in the Department of Neurosurgery, Xijing Institute of Clinical Neuroscience, Xijing Hospital, Fourth Military Medical University, China. No restrictions were placed on patients' selection with regard to histologic type of brain tumors, age, education, cognitive function or performance status. The samples were restricted to patients who required surgical intervention. Post-operation patients with scheduled radiotherapy or chemotherapy were excluded. The study was approved by the Institutional Review Board of Xijing Hospital, Fourth Military Medical University. Written informed consent was obtained from each patient or their legally authorized representative.

### The Standard Chinese version of EORTC QLQ-C30 (version 3.0)

The EORTC QLQ-C30 (version 3.0) is a 30-item questionnaire composed of multi-item scales and single items that reflect the multidimensionality of the QoL construct. It includes five functional scales (physical, role, cognitive, emotional, and social), three symptom scales (fatigue, pain, and nausea/vomiting), and a global health status/QoL scale. The remaining single items assess additional symptoms commonly reported by cancer patients (dyspnea, appetite loss, sleep disturbance, constipation, and diarrhea), as well as the perceived financial impact of the disease and treatment [11].

### Description of Procedures or Investigations undertaken

The standard Chinese version of QLQ-C30 (version 3.0) was administered at three time points: T1 (the first or the second day that patients were hospitalized in our hospital, but before surgery) to evaluate all eligible patients; T2 (1 to 2 days later after T1 but before surgery) to patients who were randomly selected from patients at T1 using computer-generated random table number; T3 (the day before discharge which is after surgery), to evaluate patients who were randomly selected from patients at T1 using computer-generated random table number. Scoring of the Chinese version of the Mini-Mental State Examination (MMSE) [12] and Karnofsky Performance Status( KPS) [13] was performed by the doctors or nurses at the time of the first administration of the QLQ-C30. MMSE and KPS are used as grouping variables.

### Statistical methods

Based on MMSE scores, patients were divided into normal cognition and abnormal cognition groups [12,14]. Concisely, MMSE scores less than 18 in illiterate patients, less than 21 in patients with elementary school education, and less than 25 in patients with more than high school education were defined as indicating abnormal cognition. Scoring of the responses to the QLQ-C30 (version 3.0) was carried out according to previously published procedures' [2,15]. The raw scores for each domain and single item were transformed to give a value between 0-100. For the five functional scales and the global health status/QoL scale, item responses were recorded so that a higher score represented a better level of functioning. For the symptom-oriented scales and items, a higher score corresponded to a severe level of symptoms. If there was no specific indication, all the data for analysis of QLQ-C30 were from T1 administration of it. The postulated scale structure was analyzed for scaling errors using multitrait scaling analysis [2]. Internal consistency reliability of multi-item scales was demonstrated by Cronbach's alpha coefficient [2]. The test-retest reliability between T1 and T2 was assessed by Pearson's Correlation Coefficient [16]. Three approaches were taken to evaluate the validity of the QLQ-C30. Correlations were determined by Pearson's Correlation Coefficient, comparison of differences within known-groups was calculated by ANOVA (for cross-sectional analysis of T1), and the responsiveness of the QLQ-C30 to changes in health status over surgery was evaluated by repeated measures ANOVA (for analysis of the change between T1 and T3) [2]. The known-groups were classified according to age, sex, cognition, KPS, and tumor types. Comparison of distribution differences between data from brain tumors and reference data from all cancer patients was done using Mann Whitney U-test [17]. The reference data is available from the EORTC Quality of Life Department. Statistical significance was defined as P < .05. All of the tests were two sided. Statistical analysis was performed using the SPSS software package, version 16.0 (SPSS Inc, Chicago, IL, USA).

## Results

There were 366 patients enrolled in the study. After excluding 6 patients who died in peri-operation period, 28 patients with pathologically confirmed non-tumors, 24 patients with significant data loss or errors, and a total of 308 patients were recruited into the current statistical analysis. Of these 308 patients, 66 (21.4%) randomly selected patients completed the questionnaire at both T1 and T2, 53 (17.2%) completed the questionnaire at both T1 and T3. All of the 308 patients were included in T1. There was no statistically significant difference concerning the demographic and clinical data between T1 population and T2 or T3 samples. The length of hospital stay was 18 ± 6 days, and the post-operative length of hospital stay was 15 ± 7 days. The clinical characteristics of all the samples were reported in Additional file 1. The sample was heterogeneous regarding to histologic type of brain tumors, cognitive function and KPS.

### Acceptability of the Questionnaire

The average time required to complete the standard Chinese version of QLQ-C30 (version 3.0) was 13-14 minutes (SD = 12 minutes). Of total 308 patients at T1, 127 patients (41.2%) were able to complete the questionnaire without assistance (self-report), 29 patients (9.4%) and 102 patients (33.1%) were able to complete the questionnaire aided by nurses or family members respectively (self-report required assistance), 32 patients (10.4%) were unable to complete the questionnaire therefore the family members reported the questionnaires (proxy-report), 18 patients (5.8%) were not recorded the reporters. Of the reasons for the patients' questionnaires whose were self report required assistance and proxy report, 26.1% was that the patients could not understand the actual language/illiterate, 25.0% was administrative failure to distribute the questionnaire to the patient, 18.6% was the patients' visual disorder, 15.4% was that the patients felt it was inconvenient or took too much time, 8.0% was that the patients felt themselves too ill, 6.9% was that the clinician or nurse felt the patient was too ill. No matter what the mode of administration was, all the questionnaires were included in the flowing analysis. In patients with abnormal cognition, the proxy-report rate (16/53) and self-report required assistance rate (24/53) were higher than that in patients with normal cognition (proxy-report rate 14/230, self-report required assistance rate 104/230). The self-report rate in patients with abnormal cognition was lower than that in patients with normal cognition (patients with abnormal cognition vs. normal cognition: 13/53 vs. 112/230) Controlling for patients' KPS, no statistically significant differences were noted in most of scales and items response dispersion (means and standard deviations), among those who were self-report, self-report required assistance and proxy-report. Since that only a very small proportion of patients with abnormal cognition had completed questionnaires by themselves (13 vs. 308), and that not every clinical trial performed cognition assessment to distinguish such patients, we did not exclude them from analysis.

### Multitrait Scaling

Item-scale correlations (corrected for overlap) exceeded the .40 criterion for item-convergent validity for seven of the nine hypothesized scales at T1. (Exceptions included one item from the emotional functioning scale and both items from the cognitive functioning scale.) The mean item-scale correlations across all nine scales were .58. There were 192 tests of item-discriminant validity (data not shown) for T1 administration. Scaling successes were noted in 97% of the cases. Taken together, the very small number of scaling errors provided strong support for the hypothesized scale structure of the QLQ-C30. The only scale that indicated consistent problems was the cognitive functioning scale.

### Descriptive Statistics and Scale Reliability

Table 1 shows the means, standard deviations, median and mode for the multi-item and single-item measures for T1 administrations of the standard Chinese version of QLQ-C30 (version 3.0). Additional file 2 shows constructed scales and items of all brain tumor patients before surgery. The full range of possible scores was observed for all measures at T1 except for the emotional functioning scale (range, 17-100) and diarrhea item (range, 0-67). Only the global health status/QoL showed normal distribution. Score distributions were roughly negative skew distribution in all functioning scales and the modal scores were frequently 100 (i.e., more patients scored toward maximum functioning). The symptom scales and single-item measures were generally positive skew distribution and the modal scores were frequently 0 (i.e., no symptoms), except that the modal scores of fatigue and mode were 33.3. As expected, constipation and diarrhea symptoms that not specifically associated with brain tumor patients were not reported frequently. Eight of the nine multi-item subscales met the minimal standards of reliability (range from .75 to .91 at T1, Cronbach's alpha coefficient >.70), except for the cognitive functioning scale (.33). (Table 1)

**Table 1:** Descriptive statistics and scale reliability of the QLQ-C30

	Items*	Mean	Median	Mode	SD	Cronbach's alpha coefficient$
Functioning scales^						
PF	1-5	81.0	86.7	100.0	23.23	0.88
RF	6, 7	79.4	100.0	100.0	28.30	0.91
EF	21-24	78.0	83.3	100.0	18.40	0.80
CF	20, 25	76.2	83.3	100.0	20.95	0.33
SF	26, 27	70.7	66.7	100.0	27.74	0.80
QL	29, 30	54.7	58.3	67.0	26.55	0.85
Symptom scales and/or items§						
FA	10, 12, 18	31.2	33.3	33.0	23.71	0.81
NV	14, 15	11.3	0.0	0.0	20.91	0.84
PA	9, 19	26.5	16.7	0.0	26.33	0.75
DY	8	10.8	0.0	0.0	19.72	
SL	11	18.5	0.0	0.0	28.60	
AP	13	19.9	0.0	0.0	28.09	
CO	16	14.5	0.0	0.0	24.19	
DI	17	2.8	0.0	0.0	10.03	
FI	28	47.1	33.3	33.0	36.33	

* Numbers correspond to the item numbers in the questionnaire.

$ Alpha values >.70 indicate adequate scale reliability.

^ Scores range from 0 to 100, with a higher score representing a higher level of functioning.

§Scores range from 0 to 100, with a higher score representing a greater degree of symptoms.

Abbreviations: AP Appetite loss, CF Cognitive functioning; CO Constipation; DI Diarrhoea; DY Dyspnoea; EF Emotional functioning; FA Fatigue; FI Financial difficulties; NV Nausea and vomiting; PA Pain; PF physical function; QL Global health status; RF role function; SF Social functioning; SD standard deviation; SL Insomnia

Pearson's correlation coefficients among all nine scales and seven items of the QLQ-C30 for both the T1 and T2 administration were statistically significant (role and emotional functioning scales p < .05, others p < .001); however, the reproducibility of the questionnaire was not satisfactory (Pearson's r < .80, physical function, global health status/QoL, fatigue, pain, finance scales' Pearson's r .50 - .79, other scales' Pearson's r .27 - .50).

### Validity of the QLQ-C30

In the following sections, the results of the three types of validity analysis outlined in the "Methods" section are presented.

#### Inter-scale correlations

Table 2 presents the correlations among the nine scales of the QLQ-C30 for both the T1 and T3 administrations. Some inter-scale correlations were statistically significant (p < .05), reflecting both the conceptual non-orthogonality of the scales and the effect of a relatively large sample size. The magnitude of these correlations is more important. The strongest correlations both before and after surgery were observed between the physical functioning and fatigue scales (ranging from -.58 to -.54). The global health status/QoL scale and fatigue scales were correlated with most of the other scales; whereas, no correlation was observed between the emotional functioning scale and the physical, role and cognitive functioning scales, between cognitive functioning scale and emotional functioning, social functioning and pain scales, between social functioning scale and cognitive functioning, nausea/vomiting, and global health status/QoL scales, between nausea/vomiting scale and role and social functioning scales. Most of other scales were correlated moderately or weakly with each other. In general, the inter-scale correlations of only a moderate or weak size indicated that, although related, they were able to reflect distinct components of the QoL construct.

**Table 2:** Correlations among the QLQ-C30 scales before and after operation

	PF	RF	EF	CF	SF	QL	FA	NV	PA
PF	.789**	.306*	0.12	.352**	.267*	0.24	-.580**	-0.24	-.427**
RF	.474**	.288*	0.21	.355**	.551**	.350**	-.366**	-0.20	-.353**
EF	-0.04	-0.01	.272*	0.21	0.10	.334**	-0.01	-.270*	-.283*
CF	.331**	0.12	0.23	.454**	0.20	.263*	-.330**	-.357**	-0.24
SF	0.18	0.18	.321**	0.22	.391**	0.22	-0.22	-0.18	-.448**
QL	.381**	.315*	.272*	.254*	0.21	.607**	-.489**	-.357**	-.616**
FA	-.540**	-.352**	-.331**	-.538**	-.322**	-.402**	.697**	.304*	.580**
NV	-.243*	0.01	0.07	0.01	-0.04	-.245*	0.20	.367**	.406**
PA	-.383**	-0.14	-0.15	-0.21	-.307*	-.346**	.296*	.316**	.610**

# Before operation under the diagonal; after operation above the diagonal. Values = Pearson's r. Negative correlations are an artifact of the scoring procedures. For the functional scales (PF, RF, CF, EF, SF, and QL), a higher score represents a higher level of functioning. For the symptom scales (FA, NV, and PA ), a higher score represents a higher level of symptoms. All correlation coefficients are statistically significant.

**. Correlation is significant at the 0.01 level (2-tailed).

*. Correlation is significant at the 0.05 level (2-tailed).

Abbreviations: CF Cognitive functioning; EF Emotional functioning; FA Fatigue; NV Nausea and vomiting; PA Pain; PF physical function; QL Global health status; RF role function; SF Social functioning

#### Clinical validity--known-groups comparisons

**Figure 1:**
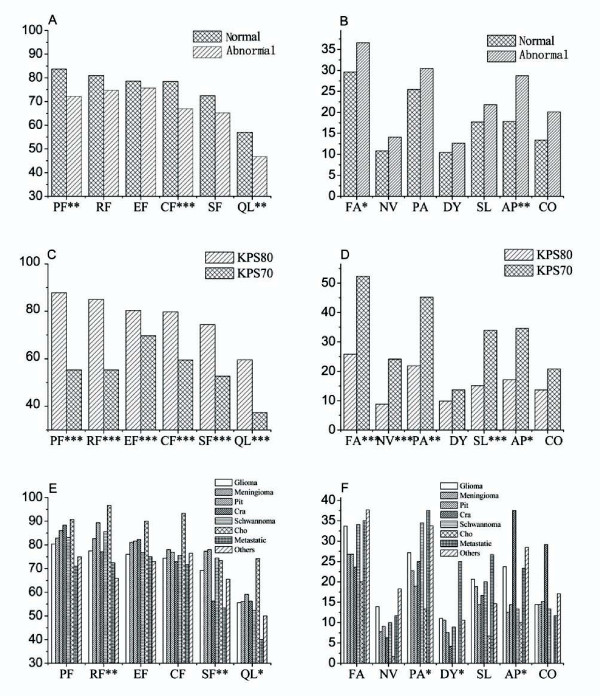
**Summary of ANOVA results of QLQ-C30 grouped by preoperative cognition (n = 297), by KPS (n = 266) and by brain tumor types (n = 304)**. ***. Correlation is significant at the .001 level (2-tailed). **. Correlation is significant at the .01 level (2-tailed). *. Correlation is significant at the .05 level (2-tailed). Abbreviations: AP appetite loss, CF cognitive functioning; Cho cholesteatoma; CO constipation; Cra craniopharyngioma; DI diarrhea; DY dyspnea; Abnormal abnormal cognition; EF emotional functioning; FA fatigue; FI financial difficulties; KPS70 KPS ≤ 70; KPS80 KPS ≥ 80; NV nausea/vomiting; Normal normal cognition; PA pain; Pit pituitary adenoma; QL global health status/quality of life; SF social functioning; SL insomnia

No statistically significant difference was observed between male and female. As expected, patients older than 50 reported worse physical (p = .003), role (p = .035) and cognitive (p = .001) functioning, worse global health status/QoL (p = .001), more symptoms of sleep (p = .025) and appetite loss (p = .021) than those younger than 50. Patients with normal cognition had better physical (p = .001) and cognitive (p < .001) functioning, better global health status/QoL (p = .007), less fatigue (p = .045) and appetite loss (p = .008) symptoms than those with abnormal cognition. (Figure 1A and 1B) Differences of the remaining scales and single-item measures, though not statistically significant, were all in the expected direction between different age groups or cognitive groups. Patients with KPS 80 - 100 showed statistically significant higher functional and lower symptom scores than those with KPS less than 80 (Figure 1C and 1D). Though the incidence of dyspnea, constipation and diarrhea didn't show statistical significance, they were reported more frequently in patients with KPS less than 80. Grouping by brain tumor types, statistically significant differences were found in role and social functioning scales, global health status/QoL, and pain, dyspnea, and appetite loss symptom scales/items (p < .05). There was a trend that patient with metastatic brain tumors reported the lowest levels for most functioning, worst global health status/QoL, and highest levels for most symptoms, followed by glioma, meningioma, pituitary adenoma and cholesteatoma. (Figure 1E and 1F) Patients with craniopharyngioma, schwannoma, and other tumors did not follow this trend because of limited number or heterogeneous populations. (The item assessing diarrhea was excluded from these analyses because of the low frequency of this symptom reported in the total sample; the item on financial impact was excluded because of the absence of explicit hypotheses regarding its association with the grouping variables.)

#### Clinical validity--responsiveness to changes over surgery

Statistically significant changes between assessments before and after surgery were only found in the physical and role functioning scales and financial difficulties item. Higher mean scores were observed in the physical and role functioning scales before surgery than after surgery (mean = 75.8 vs. 63.1, p = .003; mean = 71.7 vs. 58.8, p = .007, respectively).

### Comparison of individual brain tumor patient's score with reference value

Considering the absence of physical functioning reference data of brain tumor provided by EORTC QLQ-C30 and that surgery has a significant deleterious impact on patients' physical functioning for a short time, we could only compare our data from brain tumor patients with the reference data from all cancer patients [17]. Patients with brain tumors showed better physical, role, emotional functioning(p < .0001), worse cognitive and social functioning (p < .0001) and global health status/QoL (p < .0001), more nausea and vomiting symptoms(p < .05), less dyspnea, insomnia and diarrhea (p < .0001) than those in reference data.

## Discussion

Previous studies of the EORTC QLQ-C30 showed its validity and reliability across various countries [2], so did the standard Chinese version of the QLQ-C30 (version 3.0) in lung, gynecological, and breast cancer patients [5]. Here we presented for the first time the results of a study applying the standard Chinese version of the QLQ-C30 (version 3.0) in brain tumor patients. The questionnaire was well accepted by the patients. It cost 13-14 minutes to complete on average, and could be filled out by the patients themselves with little or no assistance in half cases. It has been extensively accepted that proxy could only be used when the information cannot be sought by self report from the patients. However, according to our results, controlling the KPS, the mode of administration (self-report, self-report required assistance and proxy-report) did not appear to influence the distributions of the scores. This was especially important to assess the HRQOL in brain tumor patients who suffered visual disorder or abnormal cognition or who were illiterate or children, thus it supported the possibility of large-scale clinical application of the questionnaire.

Multitrait scaling analysis showed that most of the item-subscale correlation coefficients met the standards of convergent and discriminant validity. Eight of the nine multi-item subscales met the minimal standards of reliability. Similar to the previous studies [5,16], the results of multitrait scaling analysis and Cronbach's alpha coefficients of the cognitive functioning subscale were questionable. There are two items to assess cognitive function (Have you had difficulty in concentrating on things, such as focusing on reading a newspaper or watching television? Have you had difficulty in remembering things?). Despite the notions that memory and concentration appear to be two distinctive aspects of cognitive functioning, a single composite index of the overall cognitive functioning scale would probably have general clinical utility. During the investigation, we found many patients with poor vision were misguided by the expression of "such as reading a newspaper or watching television". Therefore, in the future study, this explanation of concentrating should be changed. The different result between Chinese and others was caused by the language and culture difference. The test-retest reliability of the questionnaire was not so satisfactory as previous report [5]. One possible explanation was that these newly diagnosed brain tumor patients did not have much knowledge of their diseases on their initial admission but they got to know more of the severity of the diseases and bad prognosis in the following days, which was especially true and common in China. Besides, patients may have symptoms relief due to medication installed such as corticosteroids at the T2. All these may psychologically change their subjective assessments on their QoL, which was manifested by the bad reproducibility. Other time points for documenting the test-retest are needed.

The descriptive statistics indicated 100 as the mode of all the functioning scales and 0 as the mode in most of the symptom scales/items (except for fatigue scale and financial difficulty item) before surgery. These results were different from other reports whose score distributions were roughly symmetrical for the majority of the functioning scales and whose score distributions were generally well distributed for the symptom scales and single-item measures, despite that the modal scores were frequently 0 [2]. However, the distributions of our data showed some similarity to those reported by Zhao et al [5]. Compared with reference data from all cancer patients [17], brain tumor patients reported significantly better physical, role, emotional functioning, worse cognitive and social functioning and global health status/QoL, more nausea/vomiting symptoms, less dyspnoea, insomnia and diarrhea. These disparities may be attributed to the different time of administration of the questionnaire (before vs. after surgery), to different types of cancer and to different culture. Surgery was likely to temporarily bring about decreased physical, role and emotional functioning, and increased symptoms. Different diseases had different effect on HRQOL. Brain tumor patients presented more severely abnormal cognition than other cancers because the tumor directly destroyed the brain tissues which were responsible for cognition.

The majority of the functional and symptom measures were able to distinguish clearly between patients differing in terms of age (age > = 50, age < 50), cognition (normal cognition, abnormal cognition), KPS (KPS > = 80, KPS < 80), and among patients different brain tumor types. There was a trend that metastatic brain tumors were associated with worst functioning and most severe symptoms, followed by glioma, meningioma and pituitary adenoma. This tendency probably reflected the different aggressiveness of the brain tumors. The QLQ-C30 was significantly deteriorated in physical and role functioning over surgery (T1 vs.T3), while had little change in other scales and items. This may not only be attributed to poor validity of the QLQ-C30, but also because surgery could temporarily limit the patients' activity then change their physical and role functioning which need much longer time to recover after discharge, but not have direct and main effect on other functioning, symptoms, and a global health status/QoL scale. Similar phenomena were also found in other clinical trials, which showed little change of HRQOL in patients undergoing different treatments [18-21]. The factors affecting HRQOL are complicated, including age, sex, location and classification of tumor, moods, treatment strategies, expectation and experience, the relationship of patients and so on [6]. Besides, multidimensional HRQOL questionnaires containing too many items that interact with each other may counteract some effect.

For comparing the effects of treatments after surgery, the baseline HRQOL after surgery but before adjuvant treatment was more practical as many previous trials chose [21-24]. However, to compare the conservation therapies with surgery or between different surgical strategies, the baseline HRQOL before surgery is also needed. As shown previously, the HRQOL after surgery but before adjuvant treatment could predict the patients' prognosis [25]. Therefore whether the HRQOL before surgery can indicate the prognosis is also worth studying.

## Conclusions

In conclusion, our study for the first time provided a baseline the standard Chinese version QLQ-C30 (version 3.0) score in brain tumor patients before surgery. The questionnaire can effectively discriminate the known-group patients. However, its test-retest reliability and response validity need to be further investigated, and the cognitive functioning scale might need to be modified in Chinese version. Since there was no Chinese version of brain tumor specific module (such as BCM) available, the study on HRQOL in brain tumor patients only using the standard Chinese version of QLQ-C30 (version 3.0) may lose sufficient brain tumor specific information. In the future, the brain tumor specific questionnaire should be formulated for brain tumor patients in China.

## List of abbreviations

BCM: Brain Cancer Module; CF: cognitive functioning; Cho: cholesteatoma; CO: constipation; Cra: craniopharyngioma; DI: diarrhea; DY: dyspnoea; EF: emotional functioning; EORTC: European Organization for Research and Treatment of Cancer; FA: fatigue; FACT-Br: Functional Assessment of Cancer Therapy-Brain; FI: financial difficulties; HRQOL: health related quality of life; KPS: Karnofsky Performance Status; MMSE: Mini-Mental State Examination; Pit: pituitary adenoma; NV: nausea and vomiting; PA: pain; QL: global health status; QLQ-C30: Quality of Life Core Questionnaire 30; SF: social functioning; SL: insomnia.

## Competing interests

The authors declare that they have no competing interests.

## Authors' contributions

JXC and BLL participated in the design of the study, carried out the questionnaire and case records administration and drafted the manuscript. XZ and WL participated in the design of the study, carried out the questionnaire administration, and revised the manuscript. YQZ and RW participated in the design of the study and performed the statistical analysis. YQZ and HYZ conceived of the study, and participated in its design and coordination. LX and JLH participated in coordination. All authors read and approved the final manuscript.

## Pre-publication history

The pre-publication history for this paper can be accessed here:

http://www.biomedcentral.com/1471-2288/11/56/prepub

## Supplementary Material

Additional file 1 **Clinical characteristics of the study samples**. T1, the first or the second day that patients were hospitalized in our hospital, but before surgery, for all eligible patients; T2, following 1 to 2 days of T1, but before surgery, for randomly selected eligible patients; T3. the day before patients discharged (15 ± 7 days after surgery), for randomly selected eligible patients. ^# ^contains 2 chordoma, 1 fibroma sarcomatosum, 2 germinoma, 1 haemangioma, 4 hemangioblastoma, 1 hemanyiopericytoma, 1 lymphoma, 4 medulloblastoma, 1 primitive neuroectodermal tumor, 1 solitary fibrous tumor, 1 spindle cell oncocytoma, 2 trigeminal neurinoma, 20 tumor without pathological demonstration. Click here for file

Additional file 2 **All brain tumor patients constructed scales and items (before surgery)**. Abbreviations: AP Appetite loss, CF Cognitive functioning; CO Constipation; DI Diarrhoea; DY Dyspnoea; EF Emotional functioning; FA Fatigue; FI Financial difficulties; NV Nausea and vomiting; PA Pain; PF physical function; QL Global health status; RF role function; SF Social functioning; SD standard deviation; SL Insomnia. Click here for file
