# Supplementary material for: The validation of the standard Chinese version of the European Organization for Research and Treatment of Cancer Quality of Life Core Questionnaire 30 (EORTC QLQ-C30) in pre-operative patients with brain tumor in China
Source: BMC Med Res Methodol. 2011 Apr 22;11:56. doi: 10.1186/1471-2288-11-56 (PMC3112193; doi:10.1186/1471-2288-11-56)
Supplement: Additional file 1 — Clinical characteristics of the study samples. T1, the first or the second day that patients were hospitalized in our hospital, but before surgery, for all eligible patients; T2, following 1 to 2 days of T1, but before surgery, for randomly selected eligible patients; T3. the day before patients discharged (15 ± 7 days after surgery), for randomly selected eligible patients. # contains 2 chordoma, 1 fibroma sarcomatosum, 2 germinoma, 1 haemangioma, 4 hemangioblastoma, 1 hemanyiopericytoma, 1 lymphoma, 4 medulloblastoma, 1 primitive neuroectodermal tumor, 1 solitary fibrous tumor, 1 spindle cell oncocytoma, 2 trigeminal neurinoma, 20 tumor without pathological demonstration. [file 1471-2288-11-56-S1.DOC]

## Additional file 1 - Clinical characteristics of the study samples

|  | T1 | | T2 | | T3 | |
| --- | --- | --- | --- | --- | --- | --- |
|  | No. of patients | % | No. of patients | % | No. of patients | % |
| Total | 308 |  | 66 |  | 53 |  |
| Gender |  |  |  |  |  |  |
| Male | 155 | 50.3 | 30 | 45.5 | 26 | 49.1 |
| Female | 153 | 49.7 | 36 | 54.5 | 27 | 50.9 |
| Age |  |  |  |  |  |  |
| Range (Median) | 1-79(45.62) |  | 12-79(43.33) |  | 11-69(47) |  |
| Education |  |  |  |  |  |  |
| Illiterate | 26 | 8.4 | 5 | 7.6 | 5 | 9.4 |
| Elementary school | 56 | 18.2 | 18 | 27.3 | 15 | 28.3 |
| High school | 163 | 52.9 | 34 | 51.5 | 26 | 49.1 |
| College or more | 63 | 20.5 | 9 | 13.6 | 7 | 13.2 |
| KPS |  |  |  |  |  |  |
| 100 | 34 | 11 | 11 | 16.7 | 6 | 11.3 |
| 90 | 119 | 38.6 | 25 | 37.9 | 22 | 41 |
| 80 | 57 | 18.5 | 9 | 13.6 | 12 | 22.6 |
| 70 | 25 | 8.1 | 5 | 7.6 | 3 | 5.7 |
| 60 | 14 | 4.5 | 0 | 0 | 2 | 3.8 |
| 50 | 15 | 4.9 | 2 | 3.0 | 4 | 7.5 |
| <50 | 4 | 1.3 | 0 | 0 | 1 | 1.9 |
| Unknown | 40 | 13 | 14 | 21.2 | 3 | 5.7 |
| Cognitive function |  |  |  |  |  |  |
| Normal | 242 | 78.6 | 52 | 78.8 | 42 | 79.2 |
| Abnormal | 59 | 19.2 | 14 | 21.2 | 11 | 20.8 |
| Unknown | 7 | 2.2 | 0 | 0 | 0 | 0 |
| Type of brain tumors | |  |  |  |  |  |
| Glioma | 100 | 32.5 | 15 | 22.7 | 26 | 49.1 |
| Meningioma | 70 | 22.7 | 15 | 21.2 | 13 | 24.5 |
| Pituitary tumor | 44 | 14.3 | 14 | 21.2 | 2 | 3.8 |
| Craniopharyngioma | 8 | 2.6 | 2 | 3 | 1 | 1.9 |
| Schwannoma | 15 | 4.9 | 6 | 7.6 | 2 | 3.8 |
| Cholesteatoma | 10 | 3.2 | 0 | 0 | 0 | 0 |
| Metastatic tumors | 20 | 6.5 | 5 | 7.6 | 4 | 7.5 |
| Other tumors^#^ | 41 | 13.3 | 10 | 15.2 | 5 | 9.4 |
