# Supplementary material for: The validation of the standard Chinese version of the European Organization for Research and Treatment of Cancer Quality of Life Core Questionnaire 30 (EORTC QLQ-C30) in pre-operative patients with brain tumor in China
Source: BMC Med Res Methodol. 2011 Apr 22;11:56. doi: 10.1186/1471-2288-11-56 (PMC3112193; doi:10.1186/1471-2288-11-56)
Supplement: Additional file 2 — All brain tumor patients constructed scales and items (before surgery). Abbreviations: AP Appetite loss, CF Cognitive functioning; CO Constipation; DI Diarrhoea; DY Dyspnoea; EF Emotional functioning; FA Fatigue; FI Financial difficulties; NV Nausea and vomiting; PA Pain; PF physical function; QL Global health status; RF role function; SF Social functioning; SD standard deviation; SL Insomnia. [file 1471-2288-11-56-S2.DOC]

Additional table 1. All brain tumor patients constructed scales and items (before surgery)

|  | | |
| --- | --- | --- |
| PF | N | % |
| 0.0 | 6 | 1.9 |
| 6.7 | 2 | 0.6 |
| 13.3 | 5 | 1.6 |
| 20.0 | 3 | 1 |
| 26.7 | 1 | 0.3 |
| 33.3 | 2 | 0.6 |
| 40.0 | 5 | 1.6 |
| 46.7 | 5 | 1.6 |
| 53.3 | 11 | 3.6 |
| 60.0 | 7 | 2.3 |
| 66.7 | 20 | 6.5 |
| 73.3 | 22 | 7.1 |
| 80.0 | 35 | 11.4 |
| 86.7 | 36 | 11.7 |
| 91.7 | 1 | 0.3 |
| 93.3 | 57 | 18.5 |
| 100.0 | 90 | 29.2 |
| Total | 308 | 100 |
|  | | |
| RF | N | % |
| 0.0 | 14 | 4.5 |
| 16.7 | 6 | 1.9 |
| 33.3 | 19 | 6.2 |
| 50.0 | 15 | 4.9 |
| 66.7 | 58 | 18.8 |
| 83.3 | 29 | 9.4 |
| 100.0 | 167 | 54.2 |
| Total | 308 | 100 |
|  | | |
| EF | N | % |
| 16.7 | 2 | 0.6 |
| 25.0 | 1 | 0.3 |
| 33.3 | 6 | 1.9 |
| 41.7 | 11 | 3.6 |
| 50.0 | 15 | 4.9 |
| 55.6 | 1 | 0.3 |
| 58.3 | 19 | 6.2 |
| 66.7 | 45 | 14.6 |
| 75.0 | 47 | 15.3 |
| 83.3 | 51 | 16.6 |
| 91.7 | 45 | 14.6 |
| 100.0 | 65 | 21.1 |
| Total | 308 | 100 |
|  | | |
| CF | N | % |
| 0.0 | 1 | 0.3 |
| 16.7 | 4 | 1.3 |
| 33.3 | 16 | 5.2 |
| 50.0 | 35 | 11.4 |
| 66.7 | 80 | 26 |
| 83.3 | 84 | 27.3 |
| 100.0 | 88 | 28.6 |
| Total | 308 | 100 |
|  | | |
| SF | N | % |
| 0.0 | 15 | 4.9 |
| 16.7 | 4 | 1.3 |
| 33.3 | 33 | 10.7 |
| 50.0 | 29 | 9.4 |
| 66.7 | 81 | 26.3 |
| 83.3 | 51 | 16.6 |
| 100.0 | 95 | 30.8 |
| Total | 308 | 100 |
|  | | |
| QL | N | % |
| 0.0 | 14 | 4.6 |
| 8.3 | 5 | 1.6 |
| 16.7 | 18 | 5.9 |
| 25.0 | 16 | 5.2 |
| 33.3 | 31 | 10.2 |
| 41.7 | 28 | 9.2 |
| 50.0 | 39 | 12.8 |
| 58.3 | 20 | 6.6 |
| 66.7 | 49 | 16.1 |
| 75.0 | 23 | 7.5 |
| 83.3 | 31 | 10.2 |
| 91.7 | 6 | 2 |
| 100.0 | 25 | 8.2 |
| Total | 305 | 100 |
|  | | |
| FA | N | % |
| 0.0 | 50 | 16.2 |
| 11.1 | 40 | 13 |
| 22.2 | 56 | 18.2 |
| 33.3 | 68 | 22.1 |
| 44.4 | 34 | 11 |
| 55.6 | 25 | 8.1 |
| 66.7 | 12 | 3.9 |
| 77.8 | 13 | 4.2 |
| 88.9 | 6 | 1.9 |
| 100.0 | 4 | 1.3 |
| Total | 308 | 100 |
|  | | |
| NV | N | % |
| 0.0 | 207 | 67.2 |
| 16.7 | 43 | 14 |
| 33.3 | 37 | 12 |
| 50.0 | 6 | 1.9 |
| 66.7 | 8 | 2.6 |
| 100.0 | 7 | 2.3 |
| Total | 308 | 100 |
|  | | |
| PA | N | % |
| 0.0 | 92 | 29.9 |
| 16.7 | 78 | 25.3 |
| 33.3 | 77 | 25 |
| 50.0 | 18 | 5.8 |
| 66.7 | 22 | 7.1 |
| 83.3 | 10 | 3.2 |
| 100.0 | 11 | 3.6 |
| Total | 308 | 100 |
|  | | |
| DY | N | % |
| 0.0 | 224 | 73 |
| 33.3 | 71 | 23.1 |
| 66.7 | 8 | 2.6 |
| 100.0 | 4 | 1.3 |
| Total | 307 | 100 |
|  | | |
| SL | N | % |
| 0.0 | 193 | 62.7 |
| 33.3 | 78 | 25.3 |
| 66.7 | 18 | 5.8 |
| 100.0 | 19 | 6.2 |
| Total | 308 | 100 |
|  | | |
| AP | N | % |
| 0.0 | 184 | 59.7 |
| 33.3 | 76 | 24.7 |
| 66.7 | 36 | 11.7 |
| 100.0 | 12 | 3.9 |
| Total | 308 | 100 |
|  | | |
| CO | N | % |
| 0.0 | 210 | 68.2 |
| 33.3 | 69 | 22.4 |
| 66.7 | 22 | 7.1 |
| 100.0 | 7 | 2.3 |
| Total | 308 | 100 |
|  | | |
| DI | N | % |
| 0.0 | 284 | 92.2 |
| 33.3 | 22 | 7.1 |
| 66.7 | 2 | 0.6 |
| Total | 308 | 100 |
|  | | |
| FI | N | % |
| 0.0 | 76 | 24.7 |
| 33.3 | 98 | 31.8 |
| 66.7 | 65 | 21.1 |
| 100.0 | 69 | 22.4 |
| Total | 308 | 100 |
